# High frequency of WNT-activated medulloblastomas with *CTNNB1* wild type suggests a higher proportion of hereditary cases in a Latin-Iberian population

**DOI:** 10.3389/fonc.2023.1237170

**Published:** 2023-09-04

**Authors:** Daniel Antunes Moreno, Murilo Bonatelli, Augusto Perazzolo Antoniazzi, Flávia Escremim de Paula, Leticia Ferro Leal, Felipe Antônio de Oliveira Garcia, André Escremim de Paula, Gustavo Ramos Teixeira, Iara Viana Vidigal Santana, Fabiano Saggioro, Luciano Neder, Elvis Terci Valera, Carlos Alberto Scrideli, João Stavale, Suzana Maria Fleury Malheiros, Matheus Lima, Glaucia Noeli Maroso Hajj, Hernan Garcia-Rivello, Silvia Christiansen, Susana Nunes, Maria João Gil-da-Costa, Jorge Pinheiro, Flavia Delgado Martins, Carlos Almeida Junior, Bruna Minniti Mançano, Rui Manuel Reis

**Affiliations:** ^1^ Molecular Oncology Research Center, Barretos Cancer Hospital, Barretos, Brazil; ^2^ Molecular Diagnosis Laboratory, Barretos Cancer Hospital, Barretos, Brazil; ^3^ Cancer Genetics Department, Barretos Cancer Hospital, Barretos, Brazil; ^4^ Barretos School of Health Sciences Dr. Paulo Prata, Barretos Cancer Hospital, Barretos, Brazil; ^5^ Pathology Department, Barretos Cancer Hospital, Barretos, Brazil; ^6^ Department of Pathology and Forensic Medicine, University of São Paulo, Ribeirão Preto, Brazil; ^7^ Department of Pediatrics of Ribeirão Preto Medical School, University of São Paulo, Ribeirão Preto, Brazil; ^8^ Department of Neurology and Neurosurgery, Federal University of São Paulo (UNIFESP), São Paulo, Brazil; ^9^ Oncology Department, AC Camargo Hospital, São Paulo, Brazil; ^10^ Pathology Department, Italian Hospital of Buenos Aires, Buenos Aires, Argentina; ^11^ Pediatric Oncology Department, Centro Hospitalar Universitário São João, Porto, Portugal; ^12^ Department of Pathology, Centro Hospitalar Universitário São João, Porto, Portugal; ^13^ Brasília Children’s Hospital, Brasília, Brazil; ^14^ Pediatric Neurosurgery Department, Barretos Cancer Hospital, Barretos, Brazil; ^15^ Pediatric Oncology Department, Barretos Cancer Hospital, Barretos, Brazil; ^16^ ICVS/3B’s – PT Government Associate Laboratory, Braga/Guimarães, Portugal; ^17^ Life and Health Sciences Research Institute (ICVS), School of Medicine, University of Minho, Braga, Portugal

**Keywords:** medulloblastomas, WNT activated, *CTNNB1*, APC, Latin-Iberian

## Abstract

**Purpose:**

Medulloblastomas are the most common primary malignant brain tumors in children. They are divided into molecular subgroups: WNT-activated, SHH-Activated, *TP53* mutant or wild type, and non-WNT/non-SHH (Groups 3 and 4). WNT-activated medulloblastomas are usually caused by mutations in the *CTNNB1* gene (85%–90%), and most remaining cases *of CTNNB1* wild type are thought to be caused by germline mutations in *APC*. So far, the frequencies of *CTNNB1* have been reported mainly in North American and European populations. The aim of this study was to report the frequency of *CTNNB1* mutations in WNT-activated medulloblastomas in a Latin-Iberian population and correlate with their clinicopathological characteristics.

**Methods:**

A total of 266 medulloblastomas from seven different institutions from Brazil (n=211), Portugal (n=38), and Argentina (n=17) were evaluated. Following RNA and DNA isolation from formalin-fixed, paraffin-embedded (FFPE) tumor tissues, the molecular classification and *CTNNB1* mutation analysis were performed by nCounter and Sanger sequencing, respectively.

**Results:**

WNT-activated medulloblastomas accounted for 15% (40/266) of the series. We observed that 73% of WNT-activated medulloblastomas harbored *CTNNB1* mutations. *CTNNB1* wild-type cases (27%) were more prevalent in female individuals and suggested to be associated with a worse outcome. Among the *CTNNB1* wild-type cases, the available analysis of family history revealed two cases with familiar adenomatous polyposis, harboring *APC* germline variants.

**Conclusion:**

We observed a lower incidence of *CTNNB1* mutations in WNT-activated medulloblastomas in our Latin-Iberian cohort compared to frequencies previously described in other populations. Considering that *CTNNB1* wild-type cases may exhibit *APC* germline mutations, our study suggests a higher incidence (~30%) of hereditary WNT-activated medulloblastomas in the Latin-Iberian population.

## Introduction

Medulloblastomas are a group of heterogeneous embryonal tumors considered as the most common primary malignant brain tumor in children, with an annual incidence of 0.4 in 100,000 population in children and young adults aged 0–19 years (1). Histologically, medulloblastomas are divided into classic (40%–45%), desmoplastic nodular (30%–35%), anaplastic/large cell (15%–20%), and with extensive nodularity (10%) (2). These tumors are commonly divided into four molecular subgroups: WNT activated, SHH activated, Group 3, and Group 4 (2, 3). In the 2021 WHO classification, the SHH-activated group is subdivided into *TP53* wild type and *TP53* mutant, and Groups 3 and 4 are merged in a non-WNT/non-SHH subgroup (4). The standard therapy based on surgery, craniospinal irradiation, and chemotherapy may vary according to the molecular subgroup, the patient age, leptomeningeal dissemination status, and the extension of surgical resection (2).

The wingless (WNT)-activated group accounts for 10% of all medulloblastomas, is commonly observed in children older than 4 years, in an equal proportion of boys and girls, and usually shows no metastasis at diagnosis (3). This molecular subgroup is a particular entity with a distinctly better outcome in children, with more than 95% of 5-year overall survival when these children are submitted to standard therapy and displays a distinct molecular pattern of gene expression (5) and methylation profile (6).

The WNT is a conserved pathway that may induce cell proliferation and growth during development via regulation by beta-catenin, which is translocated to the nucleus for binding to transcriptional factors inducing the expression of cyclins and proto-oncogenes (7). In differentiated cells, the WNT pathway is mostly in a non-activated state, in which a disruptive complex comprised APC, AXIN, GSK3, and CK1, enabling beta-catenin phosphorylation, triggering its ubiquitination and degradation (7).

It has been reported that 85%–90% of WNT-activated medulloblastomas harbor hotspot mutations in the *CTNNB1* gene, which encodes beta catenin (8). Hotspot mutations are located at the exon 3, which corresponds to the phosphorylation site of the beta-catenin. These mutations in the exon 3 inhibits the phosphorylation of beta-catenin, triggering escape from degradation, resulting in cytoplasmatic accumulation of beta-catenin, which translocates to the nucleus inducing activation of genes involved in cell proliferation (7, 9). Most of the remaining 10%–15% of WNT-activated (*CTNNB1* wild type) tumors carry germline *APC* variants (8, 10). This later condition is observed in Turcot syndrome, when primary brain tumors such as medulloblastomas may co-occur with multiple colorectal adenomas, observed in families with familial adenomatosis polyposis (FAP) (11).

For patients with WNT-activated medulloblastomas *CTNNB1* wild type, referral for genetic risk cancer assessment and germline *APC* sequencing is recommended. It is reported that 70% of these patients will have the FAP diagnosis, triggering prevention measures such as total colectomy for the patient and germline tests to the relatives (8). Identifying these cases, patients with WNT-activated medulloblastomas and *CTNNB1* wild type have a high chance of harboring germline mutations in *APC* and should improve the patient’s treatment by differentiated surveillance and early cancer detection in patients and relatives resulting in more effective therapy (8).

The incidence of the WNT-activated *CTNNB1* wild type is currently well established for the North American and European population (8), and little is known about the frequency of these mutations in Brazilian and other Latin-Iberian countries. In the present study, we aimed to analyze the frequency of *CTNNB1* mutations in WNT-activated medulloblastomas in a large Latin Iberian medulloblastoma cohort.

## Materials and methods

### Patient cohorts

In the present retrospective study, we evaluated 266 FFPE medulloblastoma specimens collected between 2001 and 2022 from naive-treated patients from seven different institutions in Brazil, Argentina, and Portugal: Barretos Cancer Hospital, Brazil (n=119); Federal University of São Paulo UNIFESP (n=20), Brazil; AC Camargo Hospital, Brazil (n=15), Ribeirão Preto Medical School, Brazil (n=37); Child Hospital of Brasília, Brazil (n=20); Italian Hospital of Buenos Aires, Argentina (n=17); and Centro Hospitalar Universitário São João, Portugal (n=38). The frequency of molecular subgroups was previously reported in a subset of cohort^5,19^. The patient’s clinical and molecular features were collected on medical reports and stored in the Research Electronic Data Capture (RedCap). This study was approved by the institutional review board from Barretos Cancer Hospital (CAAE: 59979816.6.1001.5437). Informed consent was obtained from the patients or familiars before APC germline evaluation.

### RNA and DNA isolation

The tumor area was previously marked by an experienced pathologist, ensuring the presence of >80% of tumor cells and the absence of microvascular proliferation and necrosis. For *CTNNB1* gene analysis, DNA isolation from macrodissected formalin-fixed, paraffin-embedded (FFPE) was performed using the QIAamp DNA Mini Kit (Qiagen, Venlo, The Netherlands), following the manufacturer’s recommendations. For *APC* gene analysis, DNA was isolated from peripheral blood samples using the QIAamp DNA Blood Mini Kit (Qiagen, Venlo, The Netherlands), following the manufacturer’s instructions. The DNA quantification was performed using the NanoDropVR 2000 (Thermo Fisher Scientific, Waltham, USA) (12). The RNA isolation was performed using the deparaffinization solution (Qiagen, Venlo, The Netherlands) and the RNeasy Mini Kit (Qiagen, Venlo, The Netherlands), and the Qubit 2.0 Fluorometer (RNA HS Assay kit, Life Technologies, Thermo Fisher Scientific, Waltham, USA) was applied for RNA quantification following the manufacturer’s recommendations (13, 14).

### Molecular classification by gene expression

Gene expression assays were performed in the nCounter^®^ FLEX Analysis System available in the Molecular Oncology Research Center of Barretos Cancer Hospital (BCH) using the nCounter^®^ Elements custom panel (NanoString Technologies, Seattle, USA). The panel comprises three reference genes (*ACTB*, *GAPDH*, and *LDHA*) and 22 targets for WNT (*WIF1*, *TNC*, *GAD1*, *DKK2*, and *EMX2*), SHH (*PDLIM3*, *EYA1*, *HHIP*, *ATOH1*, and *SFRP1*), Groups 3 (*IMPG2*, *GABRAS*, *EGFL11*, *NRL*, *MAB21L2*, and *NPR3*), and Group 4 classification (*KCNA1*, *EOMES*, *KHDRBS2*, *RBM24*, *UNC5D*, and *OAS1*) as previously described (15, 16).

### *CTNNB1* and *APC* sequencing

The *CTNNB1* mutation was evaluated by Sanger sequencing using the following *CTNNB1* primers: forward, GCTGATTTGATGGAGTTGGA; reverse, GCTACTTGTTCTTGAGTGAA as reported (17). The PCR reactions were optimized using 7.2 µL of HotStarTaq Master Mix (Qiagen, Hilden, Germany), 5.6 µL of sterile nuclease-free water, 0.6 µL of magnesium chloride (5 mM), 0.3 µL of each forward and reverse primers (10 µM), and 1 µL of DNA. The PCR was performed in the Veriti 96-well thermocycler (Applied Biosystems, model 9902, Singapore) in the following conditions: 40 cycles of denaturation at 96°C for 45 s, annealing at 53°C for 45 s, and extension at 72°C for 45 s.

*APC* gene (NM_00038.5) mutations were evaluated by NGS (next-generation sequencing) in patients with a family history of colon cancer and/or polyps who have provided consent for germline molecular analysis. Library construction was carried out according to the Barretos Cancer Hospital Hereditary Rare Cancer Solution kit (Sophia Genetics, Switzerland), which includes the genes *APC*, *BRCA2*, *CEBPA*, *DICER1*, *GATA2*, *SMARCB1*, *MEN1*, *NF1*, *NF2*, *PALB2*, *PTCH1*, *PTEN*, *RB1*, *RET*, *RUNX1*, *SUFU*, *TP53*, *TSC1*, *TSC2*, and *VHL*, according to the manufacturer’s protocol. Briefly, DNA fragments were generated using an enzymatic fragmentation step. The three subsequent enzymatic steps, end-repair, A-tailing, and ligation to Illumina adapters, were performed in order to produce NGS libraries. A capture-based target enrichment was carried out on the pooled libraries. The quantitation of the final pool of libraries was performed using Qubit dsDNA HS fluorimetric assays (Life Technologies, USA). Quality control of fragment size was assessed using DNA ScreenTape analysis (4150 TapeStation System, Agilent). Sequencing was achieved with the final library concentration of 10 pM onto a 600-cycle format V3 flow cell, via Illumina MiSeq platform (Illumina, San Diego, CA, USA).

Data analysis was performed in order to detect single nucleotide variants (SNVs), insertions/deletions (indels), and copy number alterations (CNAs). Sequencing FASTQ data were analyzed by the Sophia DDM^®^ platform (Sophia Genetics, Switzerland).

The classification of each genomic variant into five different categories, namely, benign (B), likely benign (LB), variant of uncertain significance (VUS), likely pathogenic (LP), and pathogenic (P), were performed according to the American College of Medical Genetics and Genomics (ACMG) guidelines.

### *In silico* analysis of medulloblastomas molecular subgroups and *CTNNB1* mutation

To access the literature frequency of molecular subgroups and the *CTNNB1* mutation, we downloaded the clinical and molecular information on medulloblastomas from The Cancer Genome Atlas (TCGA) consortium at cBioPortal (https://www.cbioportal.org/). It included five different whole exome and genome datasets from the Pediatric Cancer Genome Project (PCGP) (whole genome, Nature 2012, n=37) (18), the Sickkids (whole genome, Nature 2016, n=46) (19), International Cancer Genome Consortium (ICGC) (whole exome, Nature 2012, n=125) (20), The German Cancer Research Center (Deutsches Krebsforschungszentrum, DKFZ) (whole exome, Nature 2017, n=491) (21), and from the Broad Institute (whole exome, Nature, 2012, n=92). We excluded duplicate registers, alterations (mutations, structural variants, and copy number) of unknown significance, and samples not classified in the molecular subgroups, totalizing 617 patients with both information regarding molecular subgroups and *CTNNB1* mutation in the WNT-activated subgroup.

### Statistical analysis

The statistical analysis was performed using the software IBM SPSS statistics version 25. The chi-square or ANOVA tests were applied for qualitative variables, and the Mann–Whitney test was applied for quantitative variables, rejecting the null hypothesis with p<0.05. For survival analyses, the Kaplan–Meier method and the log-rank test were applied.

## Results

### Higher frequency of WNT-activated medulloblastomas with *CTNNB1* wild type in Latin-Iberians population

**Figure 1:**
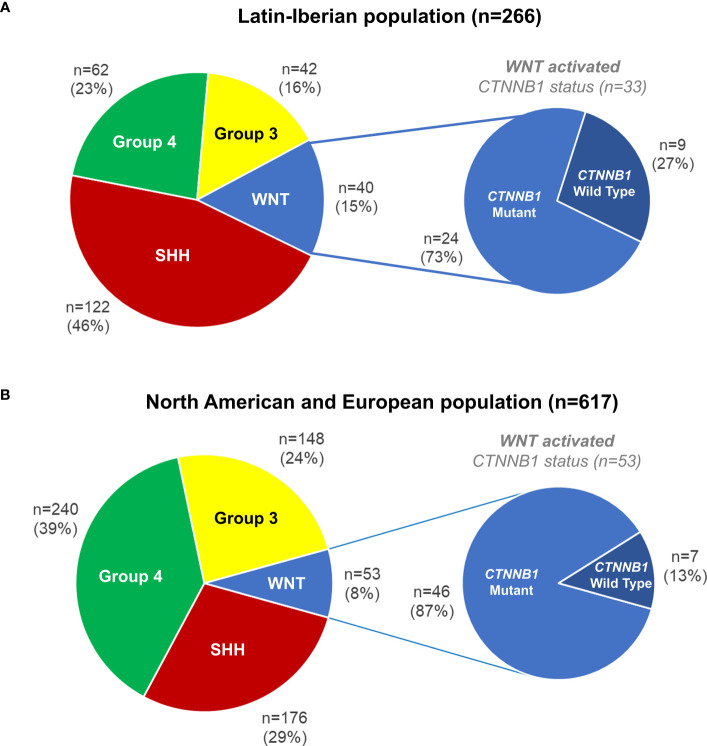
Medulloblastomas molecular subgroups and *CTNNB1* mutations in WNT-activated medulloblastomas. **(A)** Latin-Iberian population (n=266, including patients from Brazil, Portugal, and Argentina). **(B)** *In silico* analysis in medulloblastomas from the North American and European populations (n=617).

We evaluated 266 Latin-Iberian medulloblastomas from Brazil (n=212), Portugal (n=38), and Argentina (n=16). All cases were molecularly classified into the four main medulloblastomas subgroups, namely, WNT activated (n=40, 15%), SHH activated (n=122, 46%), Group 3 (n=42, 16%), and Group 4 (n=62, 23%) (**Figure 1A**). The clinicopathological features of WNT-activated medulloblastomas from the Latin-Iberian population (n=40) are outlined in detail in **Supplementary Tables 1**, **2**. Among the 40 WNT-activated, seven cases were inconclusive for *CTNNB1* mutations due to the low quantity and quality of DNA for Sanger sequencing. From the remaining 33 WNT-activated cases (24 from Brazil, seven from Portugal, and two from Argentina), we detected *CTNNB1* mutation in 24 (73%) cases, and nine (27%) medulloblastomas were *CTNNB1* wild type (**Figure 1A**).

The *in silico* analysis of medulloblastomas from the North American and European (NAM/EU) populations showed that 29% (n=176) was SHH activated, 39% (n=240) Group 4, 24% (n=148) Group 3, and 8% (n=53) WNT activated. In the WNT-activated subgroup, 87% (n=46) showed hotspot mutation in the *CTNNB1*, and 13% (n=7) were *CTNNB1* wild type (**Figure 1B**).

The frequency of WNT-activated medulloblastomas in Latin-Iberian population (**Figure 1A**) was significantly higher (15%) compared to the frequency observed in NAM/EU populations of 8% (**Figure 1B**), (p=0.000023).

### *CTNNB1* variants in the WNT-activated medulloblastomas from Latin-Iberian population

**Figure 2:**
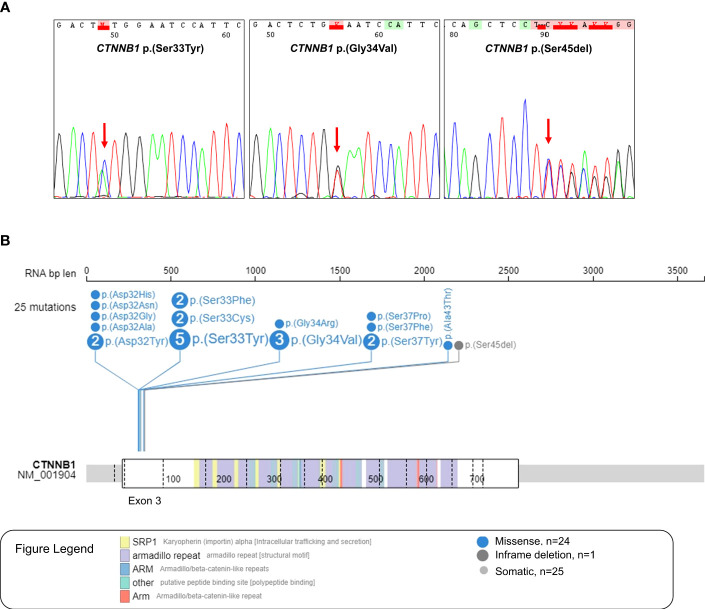
*CTNNB1* variants found in the Latin-Iberian population. **(A)** Electropherogram of Sanger sequencing of the hotspot region in the exon 3 of *CTNNB1* (chr3:41224525 + 41224751), which codes the phosphorylation site of the protein, showing three different *CTNNB1* variants: p.(Ser33Tyr), p.(Gly34Val) and p.(Ser45del). **(B)** Lollipop showing the 25 *CTNNB1* variants observed in the WNT-activated medulloblastomas from the Latin-Iberian population.

In our Latin-Iberian series of 24 *CTNNB1* mutant medulloblastomas, we found a total of 25 pathogenic variants in the hotspot region of the exon 3, being 24 missense mutations, and one in-frame deletion (**Figures 2A, B**). More detailed information about the mutational status of *CTNNB1* in WNT-activated medulloblastomas in Latin-Iberian patients is described in **Supplementary Table S2**. The most frequent *CTNNB1* variant observed was the p.(Ser33Tyr) found in five cases, followed by the p.(Gly34Val) found in three cases, and variants p.(Ser37Tyr), p.(Asp32Tyr), p.(Ser33Cys), and p.(Ser33Phe) were found in two cases each one. The remaining variants were observed only in one case (**Figure 2B**, **Supplementary Table S2**). In one case (ID88), we observed two *CTNNB1* variants, p.(Ser33Tyr) and p.(Ala43Thr) (**Supplementary Table S2**).

The *in silico* analysis of medulloblastomas from the North American and European (NAM/EU) populations reveals 47 *CTNNB1* variants in 46 WNT-activated medulloblastomas, with all variants being located in the exon 3 of the *CTNNB1* gene (**Supplementary Figure S1**). One case showed two mutations ICGC_MB113, p.(Thr41Ala) and p.(Ser33Cys). Similarly to what was observed in our series, the p.(Ser33Cys) variant was one of the most frequent detected variants (**Supplementary Figure S1**).

The frequency of WNT-activated medulloblastomas with *CTNNB1* wild type was significantly higher in Latin-Iberian population (27%) (**Figure 1A**) compared to those observed in NAM/EU populations (13%) (**Figure 1B**), (p=0.014769).

### WNT-activated medulloblastomas with *CTNNB1* wild type were prevalent in females and showed worse outcome in the Latin-Iberian population

We further associated the *CTNNB1* mutational status with our patients’ clinical–pathological features (**Table 1**). WNT-activated medulloblastoma patients with *CTNNB1* mutant showed an older median age at diagnosis of 11.3 years, compared with 10.0 years of *CTNNB1* wild type, yet not statistically significant. The *CTNNB1* wild-type cases were prevalent in female individuals (p=0.04), and no significant associations were observed regarding histology, surgery extension, and metastasis (**Table 1**).

**Table 1:** Association of *CTNNB1* status with clinicopathological features of 33 WNT-activated medulloblastomas from a Latin-Iberian population.

Features (n=33)	Variables	*CTNNB1* mutant (n=24)	*CTNNB1* wild type (n=9)	Significance
Age at diagnosis	Median (Range)	11.3 years (5.2-25.9)	10.0 years (7.0-23.5)	p = 0.41
	Pediatric (>4 and ≤ 18 years)	n = 22 (91.7%)	n = 8 (88.9%)	p = 0.81
	Adult (>18 years)	n = 2 (8.3%)	n = 1 (11.1%)	p = 0.83
Gender	Male	12 (50.0%)	n = 1 (11.1%)	**p = 0.04**
	Female	12 (50.0%)	n = 8 (88.9%)	
Histology	Classic	18 (94.7%)	6 (85.7%)	p = 0.85
	Extensive nodularity	0 (0.0%)	1 (14.3%)	
	Anaplastic / large cells	1 (5.3%)	0 (0.0%)	
	Missing	5	2	
Surgery Extension	Total	12 (70.6%)	2 (40.0%)	p = 0.19
	Partial	5 (29.4%)	3 (60.0%)	
	Missing	7	4	
Metastasis at diagnosis	No	17 (89.5%)	7 (87.5%)	p = 0.85
	Yes	2 (10.5%)	1 (12.5%)	
	Missing	5	1	
Status*	Alive	20 (100.0%)	5 (71.4%)	**p = 0.01**
	Deceased by cancer	0 (0.0%)	2 (28.6%)	
	Deceased by other reasons	1	2	
	Missing	3	0	
Follow-up	Median (months)	54.6 (0.03-161.66)	42.1 (1.94-248.13)	p = 0.85
	Missing	2	2	

*In the statistical analysis for status, was included only Deceased by cancer, and 6 cases were not evaluated due to lack of available clinical or died by other reasons.

The bolded entries in **Table 1** indicate significant differences (p < 0.05).

Despite not being statistically significant, we observed that *the CTNNB1* mutant had a better outcome, with a 54.6-month median follow-up, compared with 42.1 months in wild-type cases (**Table 1**). We observed that 28.6% (2/7) of *CTNNB1* wild-type patients died of cancer, contrasting with *CTNNB1* mutant cases, where any patient died of cancer (p=0.01) (**Table 1**). Three *CTNNB1* mutants were lost to follow-up (missing: ID97, 197, and 260), and additionally, three patients died due to other reasons (ID94, ID95, and ID96), thus were not included in the survival analysis (**Supplementary Table S2**).

**Figure 3:**
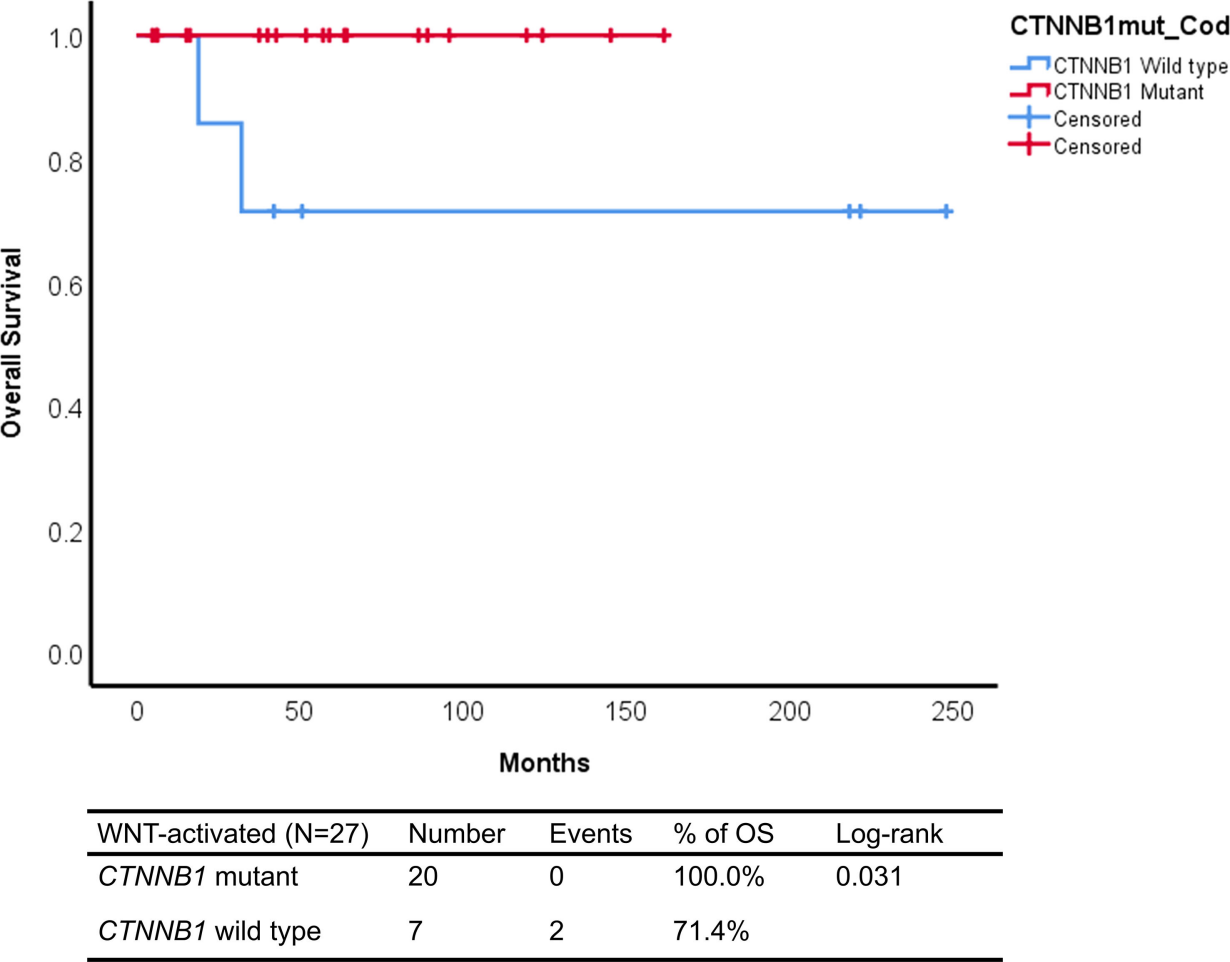
Kaplan–Meier curves of *CTNNB1* mutant and *CTNNB1* wild-type WNT-activated medulloblastomas from Latin-Iberian population.

The Kaplan–Meier analysis showed that patients with WNT-activated medulloblastomas *CTNNB1* wild type had worse outcome, with 71.4% of overall survival compared to 100% of *CTNNB1* mutant cases (log rank: p=0.031) (**Figure 3**).

### *APC* germline mutation associated with WNT-activated medulloblastomas

**Figure 4:**
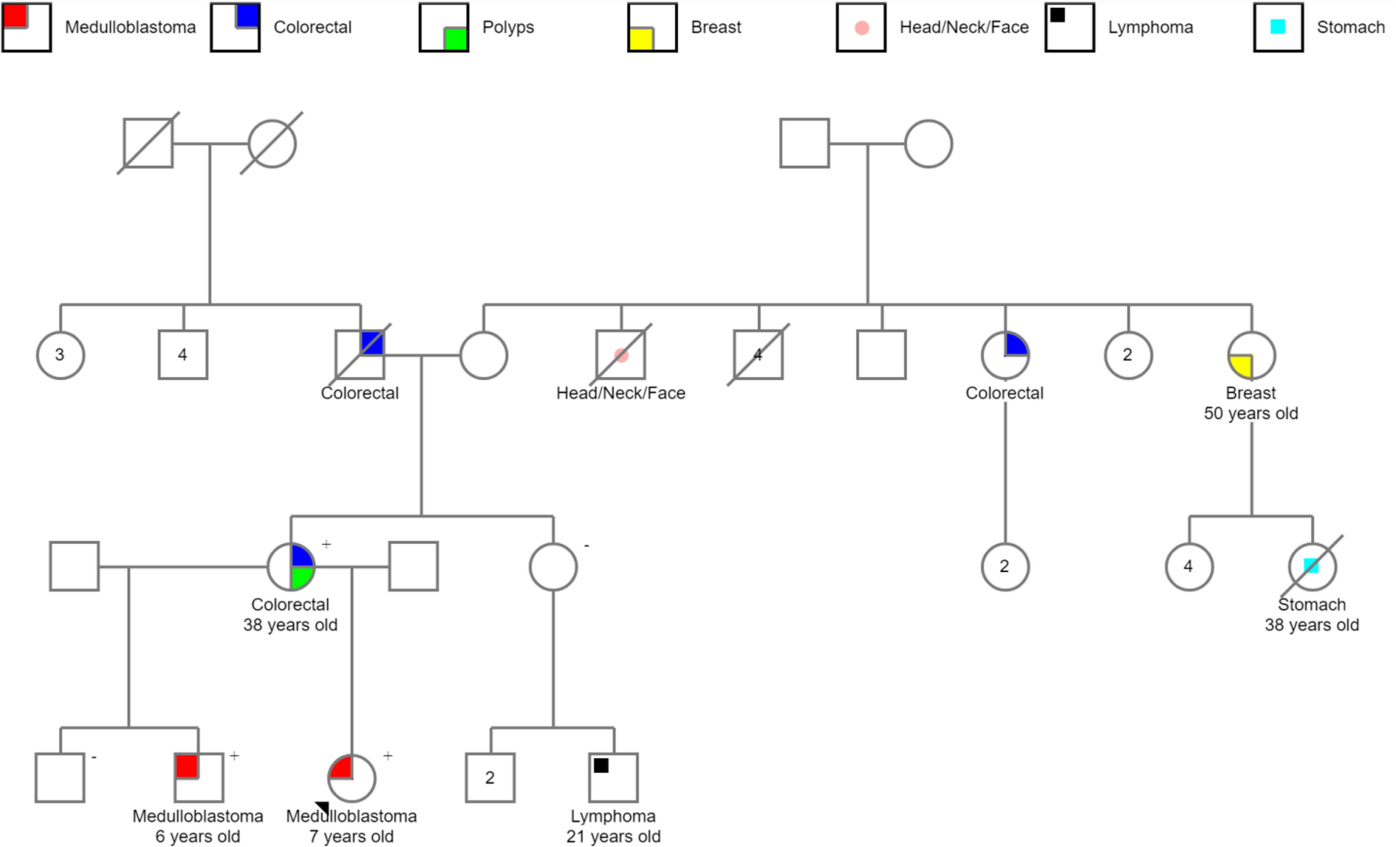
Pedigree of familial adenomatous polyposis showing the occurrence of APC-associated WNT-activated medulloblastomas in siblings. The *APC* germline mutation was tested, and the “+” indicates *APC* mutation and “−,” *APC* wild type.

We found nine WNT-activated medulloblastomas *CTNNB1* wild type in our Latin-Iberian population. The analysis of the clinical records available showed the existence of two reports of familial adenomatous polyposis, one from Barretos Cancer Hospital (Brazil) (**Figure 4**) and one from Centro Hospitalar Universitário São João (Portugal).

Of note, the Brazilian case, a germline *APC* c.3183_3147delACAAA-p.(Gln1062Ter) pathogenic variant was detected. The proband of this family is a 7-year-old girl diagnosed with medulloblastoma; her brother was also diagnosed with medulloblastoma when he was 6 years old. Their mother had approximately 100 polyps and developed colorectal cancer at the age of 38, fulfilling the criteria of FAP (also known as Turcot syndrome type 2) (**Figure 4**).

The Portuguese patient harbored a c.3183_3187delACAAA-p.(Gln1062Ter) *APC* germline variant, similarly to the variant detected in the Brazilian family. The patient was a 9.7-year-old girl diagnosed with WNT-activated, *CTNNB1*-wild type medulloblastoma. At 24 years old, she presented gastrointestinal tumors with hepatic metastasis. Currently, she is 30 years old, and in total remission of the brain tumor.

## Discussion

The origin of the WNT-activated medulloblastomas is attributed to molecular alterations that promote nuclear accumulation of beta-catenin products, inducing cell proliferation and tumor growth (22). Landmark genomic studies have shown that 97% of WNT-driven medulloblastomas can be explained by somatic mutations in the *CTNNB1* gene (~90%) and germline mutations in *APC* (~10%), which are mutually exclusive (8, 10).

In the present study, we report for the first time, the frequency of *CTNNB1* mutations in WNT-activated medulloblastomas in a large cohort of Latin-Iberian patients. The WNT-activated medulloblastoma subgroup in our Latin-Iberian population was of 15% (n=40), which is higher than those reported in North American and European populations (7%–10%) (2). A higher proportion of WNT-activated medulloblastomas were also described in previous Brazilian cohorts, 16.1% (24/149) (5) and 27% (24/92) (23). Another notable distinction observed in our cohort was the higher frequency of 46% for SHH-activated medulloblastomas. A potential reason for these distinct frequencies observed may lay in the methodologies used (24). In our study, we used a robust 22-gene panel assay by nCounter (25). However, DNA methylation assays have been the most recent approach recommended for medulloblastoma classification (26). A comparison study among methodologies showed that up to 10% of WNT medulloblastomas previously determined by nCounter were further classified as high-grade neuroepithelial tumor with BCOR alteration and anaplastic pilocytic astrocytoma by the DNA methylation assay (24). Further studies are needed to understand whether there is methodological issue, a hospital-based bias selection, or true epidemiological differences of medulloblastoma molecular subgroups among populations.

Among the 40 WNT-activated medulloblastomas included in the present study, we successfully evaluated *CTNNB1* mutations in 33 cases and found 73% (24/33) of *CTNNB1*-mutated cases and 27% (9/33) *CTNNB1* wild type. Of note, our frequency of *CTNNB1* wild-type WNT-activated medulloblastomas is significantly higher than that reported in North American and European populations, varying from 6.8% (8) to 13% (*in silico* analysis). Waszak and colleagues performed whole exome sequencing in 66 WNT-activated medulloblastomas and found somatic *CTNNB1* mutations in 89.4% and *CTNNB1* wild type in 10.6% of cases^8^. A recent German study evaluated a large cohort of 191 WNT-activated medulloblastomas and reported 92.2% (176/191) *CTNNB1* mutants and 7.8% (15/191) wild-type cases (10). Our *in-silico* analysis of *CTNNB1* mutations at cBioPortal showed that 13% (7/53) of WNT-activated medulloblastomas are *CTNNB1* wild type.

The reason for this discrepancy in the frequency of *CTNNB1* mutations in different populations needs to be clarified. In the present study, we performed Sanger sequencing of the exon 3 of the *CTNNB1* gene, and the studies mentioned above used whole genome or whole exome sequencing (10, 27). Nevertheless, all mutations reported by NGS were located in the exon 3, covered by our Sanger sequencing assay. Moreover, the *CTNNB1* variants identified in our Latin-Iberian study is very similar to the variants reported in the North American and European populations.

We found that patients’ *CTNNB1* wild type was more frequent in female individuals and was associated with a worse outcome. Nevertheless, caution should be taken, since these findings are based on a few patients and in a very heterogeneously treated population. Our findings contrast with other North American and European studies that did not observe any association of *CTNNB1* status with WNT-activated medulloblastoma clinicopathological features (8, 10). Therefore, further studies with more extensive series from non-European populations are warranted to explore the clinical impact of *CTNNB1* mutations in WNT-activated medulloblastomas. Moreover, other molecular features, namely, somatic alterations in *TP53*, *OTX2*, and monosomy for chromosome 6, have been associated with prognosis in WNT-activated medulloblastomas (10, 28). Additionally, addressing these alterations is needed to fully characterize the present Latin-Iberian cohort.

Considering that in WNT-activated medulloblastomas, *CTNNB1* wild-type cases can harbor *APC* germline mutations, our study suggests that up to 27% of Latin-Iberian WNT-activated medulloblastomas can occur in the context of FAP, contrasting with the reported approximately 10% in North American and European populations. The rates of germline mutations can vary between different populations (29). A cross-sectional study evaluated the frequencies of germline mutations in *APC* in more than six thousand individuals with a history of colorectal cancer in their families. It showed that *the APC* mutation rate was higher in Asians than in Caucasians (Western/Northern European, Central/Eastern European, and Ashkenazi ancestry), African American, and others (Latin American/Caribbean, Near/Middle Eastern, and Native American) (30).

Disparities in genomic studies due to the under-representation of some populations, such as from South America, were previously demonstrated (31). Consequently, genomic data from North America and the European population may only partially capture the genetic variability range in low- and middle-income countries (32). In this context, the data from our current study may contribute to the characterization of WNT-activated medulloblastomas in a poorly explored population.

It is estimated that germline *APC* mutations are associated with a 92 times higher risk for developing medulloblastomas than in the general population (11). Medulloblastoma was reported to be the most common brain tumor (79%, 11/14) observed in families with FAP^10^. Waszak and coworkers reported that all *APC* mutation carriers with available medical records (n=4) had a family history of FAP and associated cancers. Additional malignancies were observed in three patients with *APC* germline mutations (8).

Based on the available medical records, the present study identified two families fulfilling the FAP criteria. Due to the study’s retrospective nature, several clinical records are very omissive in the familiar history description, not allowing for an accurate assessment of the putative hereditary nature of the *CTNNB1* wild-type cases. Nevertheless, an active search of the *CTNNB1* wild-type patients will be done, and genetic counseling and potential confirmation of its germline nature will be offered. Importantly, the possibility of a new or founder *APC* mutation cannot be entirely ruled out (33). These data demonstrate the importance of *CTNNB1* genetic testing and should indicate that patients with WNT-activated medulloblastomas *CTNNB1* wild type must be monitored by a multidisciplinary team, due to possible hereditary nature of the disease and propensity to develop other tumor types.

Interestingly, one of our FAP exhibited a rare co-occurrence of medulloblastomas in siblings. The *APC* variant identified in this family, p.(Gln1062Ter), has been previously detected in families with classic FAP (34–36) and reported founder in Spanish and Greek populations (27, 37). This variant is located in a region of the *APC* gene associated with a higher risk of developing extracolonic tumors (38), which may explain the development of the two reported medulloblastomas. To our knowledge, only one case of siblings with *APC*-associated WNT-activated medulloblastomas was reported, involving an 11-year-old girl and her 19-year-old brother exhibiting both *APC* germline mutation p.(R213*) (10).

In conclusion, the reported higher incidence of *CTNNB1* wild type in our Latin-Iberian patients may be associated with a worse outcome and suggests a higher prevalence of hereditary WNT-activated medulloblastomas in this poorly characterized population. We also reported a rare case of siblings with WNT-activated medulloblastomas associated with *APC* germline mutation in a South American patient.

## Data availability statement

The datasets presented in this study can be found in online repositories. The names of the repository/repositories and accession number(s) can be found in the article/**Supplementary Material**.

## Ethics statement

This study was approved by the institutional review board from Barretos Cancer Hospital (CAAE: 59979816.6.1001.5437). Informed consent was obtained from the patients or familiars before APC germline evaluation.

## Author contributions

DM: data collection, experimental procedures, data analysis, and manuscript writing; MB: experimental procedures, results discussion, and manuscript review. AA: oncogenetic analysis, results discussion, manuscript writing, and review. FP: experimental procedures, results discussion, and manuscript review. LL: experimental procedures, manuscript writing, and review. FG: experimental analysis, medical reports review, manuscript writing, and review. AP: experimental procedures, manuscript writing, and review. GT: pathological review of tumor samples from Barreto’s cancer hospital, Brazil, results discussion, manuscript review. IS: pathological review of tumor samples from Barreto’s cancer hospital, Brazil, results discussion, manuscript review. FS: pathological review of tumor, results discussion, and manuscript review. LN: pathological review of tumor, results discussion, and manuscript review. EV: patient’s clinical data collection, and manuscript review. CS: patient’s clinical data collection, and manuscript review. JS: patient’s clinical data collection and manuscript review. SM: patient’s clinical data collection, and manuscript review; ML: Patient’s clinical data collection and manuscript review; GH: patient’s clinical data collection and manuscript review; HG-R: Pathological review of tumor and patient’s clinical data collection, manuscript review. SC: pathological review of the tumor and patient’s clinical data collection and manuscript review. SN: patient’s clinical data collection and manuscript review. MG-d-C: patient’s clinical data colection and manuscript review. JP: pathological review of tumor and manuscript review. FM: patient’s clinical data colection and manuscript review. CJ: medical reports analysis, patients’ treatment protocol review, and manuscript review. BM: medical reports analysis, patients’ treatment protocol review, and manuscript review. RR: supervisor and project coordinator, results discussion, and manuscript writing. All authors contributed to the article and approved the submitted version.

## Supplementary material

Supplementary Figure 1 Lollipop showing the 47 *CTNNB1* variants observed in the 46 WNT-activated medulloblastomas from the North American and European populations. Data collected at cBioPortal.
