# Supplementary figures and images for: High frequency of WNT-activated medulloblastomas with CTNNB1 wild type suggests a higher proportion of hereditary cases in a Latin-Iberian population
Source: Front Oncol. 2023 Sep 4;13:1237170. doi: 10.3389/fonc.2023.1237170 (PMC10513896; doi:10.3389/fonc.2023.1237170)

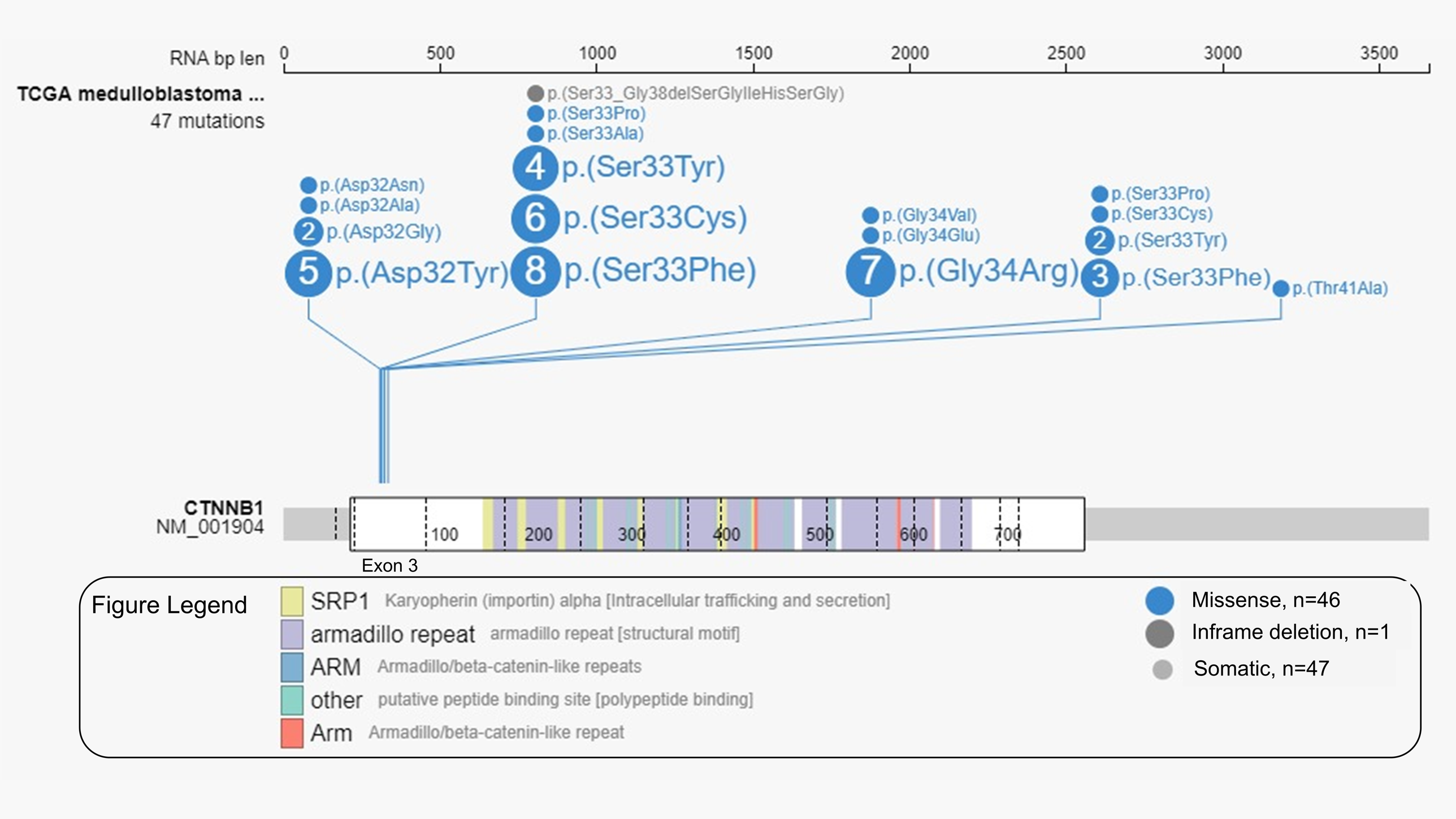

Supplement: Supplementary Figure 1 — Lollipop showing the 47 CTNNB1 variants observed in the 46 WNT-activated medulloblastomas from the North American and European populations. Data collected at cBioPortal. [file Image_1.tif]
