# Supplementary material for: High frequency of WNT-activated medulloblastomas with CTNNB1 wild type suggests a higher proportion of hereditary cases in a Latin-Iberian population
Source: Front Oncol. 2023 Sep 4;13:1237170. doi: 10.3389/fonc.2023.1237170 (PMC10513896; doi:10.3389/fonc.2023.1237170)
Supplement: Supplementary file 2 [file Table_1.docx]

**Supplementary Table 1.** Summary of clinicopathological features of WNT-activated medulloblastomas from Latin-Iberian population.

| **WNT-activated medulloblastomas from Latin-Iberian population (n=40)** | | | |
| --- | --- | --- | --- |
|  | **Characteristic** | **Number (% of valid cases)** | |
| **Age** | Median: 12.5 years (5.2 - 25.9) | |  |
| **Age group** | Infants (<4 years) | | N = 0 (0.0%) |
|  | Pediatric (>4 and ≤ 18 years) | | n = 36 (90.0%) |
|  | Adult (>18 years) | | n = 4 (10.0%) |
|  |  | |  |
| **Gender (1.7:1)** | Female | | n = 25 (62.5%) |
|  | Male | | n = 15 (37.5%) |
|  |  | |  |
| **Histologic subtype** | Classic | | n = 29 (93.5%) |
|  | Anaplastic / large cells | | n = 1 (3.2%) |
|  | Extensive nodularity | | n = 1 (3.2%) |
|  | Missing | | n = 9 |
|  |  | |  |
| **Metastasis** | No | | n = 31 (88.6%) |
|  | Yes | | n = 4 (11.4%) |
|  | Missing | | n = 5 |
|  |  | |  |
| **Surgery** | Total Resection | | n = 18 (62.1%) |
|  | Partial Resection | | n = 11 (37.9%) |
|  | Biopsy | | n = 0 (0.0%) |
|  | Missing | | n = 11 |
|  |  | |  |
| **Follow-up** | Median | | 54 months |
| **Status** | Alive | | n = 29 (82.9%) |
|  | Deceased by cancer | | n = 2 (5.7%) |
|  | Deceased by other reasons | | n = 4 (11.4%) |
|  | Missing | | n = 5 |
|  |  | |  |
| ***CTNNB1*** | Mutated | | n = 24 (73%) |
|  | Wild Type | | n = 9 (27%) |
|  | Missing | | n = 7 |
