# Supplementary material for: High frequency of WNT-activated medulloblastomas with CTNNB1 wild type suggests a higher proportion of hereditary cases in a Latin-Iberian population
Source: Front Oncol. 2023 Sep 4;13:1237170. doi: 10.3389/fonc.2023.1237170 (PMC10513896; doi:10.3389/fonc.2023.1237170)
Supplement: Supplementary file 3 [file Table_2.docx]

**Supplementary Table 2.** Clinicopathological and mutational status of *CTNNB1* in WNT-activated medulloblastomas from Latin-Iberian population.

| **ID** | **Sex** | **Age** | **Histology** | **Surgical Resection** | **Metastasis** | **Follow-up (months)** | **Status** | ***CTNNB1* mutation** |
| --- | --- | --- | --- | --- | --- | --- | --- | --- |
| 88 | F | 5-10 | Classic | Missing | No | 95.89 | Alive | p.(Ser33Tyr) and p.(Ala43Thr) |
| 89 | M | 5-10 | Classic | Total | No | 63.73 | Alive | p.(Asp32Tyr) |
| 97 | F | 5-10 | Classic | Total | No | 11.99 | Missing | p.(Ser33Cys) |
| 100 | F | >18 | Classic | Missing | No | 57.2 | Alive | p.(Ser33Phe) |
| 101 | F | 5-10 | Classic | Partial | No | 89.19 | Alive | p.(Gly34Val) |
| 103 | M | >18 | Classic | Partial | No | 0.03 | *Dead | p.(Ser37Pro) |
| 107 | M | >18 | Classic | Partial | No | 59.13 | Alive | p.(Ser33Phe) |
| 147 | F | 11-17 | Classic | Total | No | 64.45 | Alive | p.(Ser33Tyr) |
| 166 | M | 5-10 | Anaplastic / large cells | Total | No | 42.87 | Alive | p.(Gly34Val) |
| 174 | F | 5-10 | Classic | Total | No | 40.21 | Alive | p.(Ser37Tyr) |
| 175 | M | 5-10 | Classic | Total | No | 37.65 | Alive | p.(Ser37Tyr) |
| 191 | F | 11-17 | Classic | Total | Missing | 15.37 | Alive | p.(Asp32Ala) |
| 197 | F | 11-17 | missing | Missing | No | Missing | Missing | p.(Asp32Tyr) |
| 220 | M | 11-17 | Classic | Total | Yes | 4.89 | Alive | p.(Ser33Tyr) |
| 226 | F | 5-10 | Classic | Total | Yes | 119.55 | Alive | p.(Asp32Asn) |
| 247 | F | 5-10 | Classic | Partial | No | 161.66 | Alive | p.(Ser33Tyr) |
| 252 | M | 11-17 | Classic | Total | No | 124.41 | Alive | p.(Ser37Phe) |
| 260 | M | 11-17 | Classic | Total | No | Missing | Missing | p.(Ser45del) |
| 263 | M | 11-17 | Classic | Partial | No | 86.47 | Alive | p.(Gly34Arg) |
| 276 | F | 11-17 | Missing | Total | No | 145.34 | Alive | p.(Ser33Cys) |
| 286 | F | 5-10 | Classic | Total | No | 52.00 | Alive | p.(Asp32His) |
| 310 | M | 11-17 | Missing | Missing | Missing | 16.43 | Alive | p.(Ser33Tyr) |
| 317 | M | 5-10 | Missing | Missing | Missing | 6.47 | Alive | p.(Gly34Val) |
| 322 | M | 11-17 | Missing | Missing | Missing | 6.01 | Alive | p.(Asp32Gly) |
| 90 | F | 5-10 | Classic | Partial | No | 50.79 | Alive | No |
| 94 | F | >18 | Classic | Total | No | 1.94 | *Dead | No |
| 96 | F | 5-10 | Classic | Partial | No | 10.28 | *Dead | No |
| 98 | F | 11-17 | Classic | Missing | Missing | 221.81 | Alive | No |
| 99 | F | 5-10 | Extensive nodularity | Missing | No | 32.09 | Dead | No |
| 102 | F | 5-10 | Classic | Missing | No | 8.48 | Dead | No |
| 278 | F | 11-17 | Missing | Total | No | 218.46 | Alive | No |
| 280 | F | 5-10 | Missing | Missing | No | 248.13 | Alive | No |
| 292 | M | 11-17 | Classic | Partial | Yes | 42.15 | Alive | No |
| 92 | M | 11-17 | Classic | Total | No | 44.75 | Alive | #NA |
| 254 | M | 5-10 | Missing | Total | Yes | 111.2 | Alive | #NA |
| 125 | F | 11-17 | Missing | Partial | No | 95.53 | Alive | #NA |
| 86 | F | 11-17 | Classic | Total | No | 123.29 | Alive | Inconclusive |
| 91 | F | 11-17 | Classic | Total | No | 132.33 | Alive | Inconclusive |
| 95 | F | >18 | Classic | Partial | No | 1.41 | *Dead | Inconclusive |
| 291 | F | 11-17 | Classic | Partial | No | 118.04 | Alive | Inconclusive |

*Death by other reasons (ID94, ID95 and ID96); #NA= Not Analyzed (no material available for analysis)
